# On Lies and Hard Truths

**DOI:** 10.3389/fpsyg.2021.687913

**Published:** 2021-07-07

**Authors:** Sascha Behnk, Ernesto Reuben

**Affiliations:** ^1^Department of Banking and Finance, University of Zurich, Zurich, Switzerland; ^2^IU International University of Applied Sciences, Erfurt, Germany; ^3^Social Science Division, New York University Abu Dhabi, Abu Dhabi, United Arab Emirates; ^4^Center for Behavioral Institutional Design, New York University Abu Dhabi, Abu Dhabi, United Arab Emirates

**Keywords:** lying, hard truth, sender-receiver games, social image, antisocial behavior

## Abstract

We run an experimental study using sender-receiver games to evaluate how senders' willingness to lie to others compares to their willingness to tell hard truths, i.e., promote an outcome that the sender knows is unfair to the receiver without explicitly lying. Unlike in previous work on lying when it has consequences, we do not find that antisocial behavior is less frequent when it involves lying than when it does not. In fact, we find the opposite result in the setting where there is social contact between senders and receivers, and receivers have enough information to judge whether they have been treated unfairly. In this setting, we find that senders prefer to hide behind a lie and implement the antisocial outcome by being dishonest rather than by telling the truth. These results are consistent with social image costs depending on the social proximity between senders and receivers, especially when receivers can judge the kindness of the senders' actions.

## 1. Introduction

An extensive body of literature has shown that individuals face psychological costs from lying to others and has identified various factors moderating these costs^1^. A crucial moderator for lying behavior are individuals' social image concerns (see, Bénabou and Tirole, 2006; Andreoni and Bernheim, 2009). For example, Khalmetski and Sliwka (2019) developed a model that predicts partial lying due to image costs in the Fischbacher and Föllmi-Heusi (2013) die-rolling paradigm. Their findings indicate that individuals with a strong reputation sensitivity cover their lies by not always lying maximally and, thus, reducing their social image costs. Other studies substantiate these findings in different versions of the die-rolling paradigm by showing that social image costs mediate lying costs (Gneezy et al., 2018; Bašic and Quercia, 2020). While this research shows that image costs provide a strong motivation not to lie, the literature has not thoroughly investigated the impact of social image costs in settings where lying has negative consequences for others, but the alternative to lying is to be honestly antisocial. In this study, we explore circumstances under which implementing an antisocial outcome through a lie can be preferred to implementing it without lying.

The seminal experimental study on the interplay of lying behavior and its consequences is Gneezy (2005). This study shows that individuals show a lower willingness to act antisocially toward another person when an action involves lying compared to when it does not^2^. To establish this result, Gneezy (2005) compares decisions in a sender-receiver game with those in a dictator game. In the sender-receiver game, players face two options: one pays more to the sender while the other pays more to the receiver. Receivers pick the option that determines both players' earnings, but they have no information about the payoff structure. Their only information stems from a message sent by the sender. Unbeknownst to the receiver, senders can send either (i) a dishonest message that tricks the receiver into believing that the option that favors the sender is their best choice or (ii) a truthful message that reveals the option that favors the receiver. In the dictator game, players face the same payoff structure and information as in the sender-receiver game. However, dictators simply choose the option to determine the earnings of both players. Gneezy (2005) finds that senders send the dishonest message less frequently than dictators choose the option that favors them.

Although dictators can implement the same outcomes as senders in the sender-receiver game, these games vary in meaningful ways. First, in the dictator game, receivers are not actively involved in the decision-making. Hence, in contrast to senders, dictators are not intentionally influencing their counterpart's payoff-relevant behavior. Second, the framing of the action changes. Dictators are making a choice that directly determines payoffs, while senders are simply transmitting information. In the latter case, there is more moral wiggle room since senders can convince themselves that receivers chose to listen to them and are therefore responsible for the outcome^3^. To wit, the receiver in the sender-receiver game is arguably more salient than the receiver in the dictator game, which can imply that social image costs play a more prominent role in the former than the latter. These dissimilarities make it hard to attribute the difference between the dictators' and senders' choices solely to the fact that the senders' choice involves lying.

Instead of a dictator game, we use a modified sender-receiver game as the no-lying baseline. More specifically, in this *Hard Truth* sender-receiver game, receivers are not passive since their choice determines both players' payment. The difference is that senders can only send messages that truthfully reveal the earnings of the receiver. In other words, we allow for a similar interaction between players (information transmission) as well as active decision-making by the receiver and only vary the type of messages available to the sender. This design allows us to make a more direct evaluation of the effect of lying in otherwise identical settings.

We further study the difference in the senders' willingness to tell a lie vs. a hard truth by varying the prominence of social image costs. More specifically, in addition to the anonymous (computerized) message transmission in our *Baseline* treatment, we run a *Face to Face* treatment where senders personally deliver the message to the receiver. Although senders' identity is not revealed, social contact with the receiver presumably increases the senders' social image costs^4^. Finally, we run a *Face to Face & Information* treatment where, in addition to personal delivery of the message, receivers are fully informed of the game's payoff distribution^5^. This information introduces an interesting dimension to the game. In this treatment, there is no ambiguity of the sender's intentions as receivers know how much money they earn if the sender reveals the prosocial option or the antisocial option^6^. Therefore, the difference between a dishonest message and a hard truth is that in the latter, receivers learn whether the sender treated them unfairly the moment they receive the message. By contrast, if the message is dishonest, receivers learn whether the sender treated them unfairly (and the fact that the sender lied) later when they are told their earnings. In other words, a dishonest message allows senders to mask their actions at the moment of personal contact. If personal contact heightens the importance of social image costs, this treatment allows us to study a setting where lying might actually imply smaller image costs than telling a hard truth.

## 2. Materials and Methods

### 2.1. Experimental Design

In the experiment, participants are randomly matched into pairs to play a sender-receiver game. In each pair, one participant is assigned the role of the *sender* and the other the role of the *receiver*.

The receiver determines both participants' earnings by choosing one of ten options. There is one prosocial option that pays €10 to each participant, one antisocial option that pays the sender €17 minus an amount *x*∈[€0, €6.5] and €3 to the receiver, and eight Pareto-dominated options that pay €4 to the sender and €0 to the receiver. At the beginning of the game, the computer randomly labels the ten options with a unique letter ranging from A to J. Only the sender knows how each option is labeled. Table 1 is an example of a letter assignment and how this information is presented to the sender.

**Table 1:** Example payoff table in the sender-receiver games (amounts in euros).

**Option**	**A**	**B**	**C**	**D**	**E**	**F**	**G**	**H**	**I**	**J**
Sender	4	4	10	4	17 − *x*	4	4	4	4	4
Receiver	0	0	10	0	3	0	0	0	0	0

The task of the sender is to transmit a message to the receiver. There are two available messages. In the *Lying* condition, the first message, Message I, accurately reveals the label of the prosocial option and reads, “Option [letter paying the receiver €10] will earn you *more money than the other options*, 10 euros.” The second message, Message II, is dishonest in that it reveals the label of the antisocial option but claims it is the best option for the receiver: “Option [letter paying the receiver €3] will earn you *more money than the other options*, 3 euros.” In the *Hard Truth* condition, Message I and Message II simply indicate the amount the receiver will earn. Namely, Message I reads “Option [letter paying the receiver €10] will earn you 10 euros,” while Message II reads “Option [letter paying the receiver €3] will earn you 3 euros^7^.”

Our aim with these sender-receiver games is for us to be able to inform receivers of the payoff structure while maintaining the senders' incentive to reveal their preferences (in contrast to Gneezy, 2005; see Sutter, 2009). In other words, we selected the payoffs and number of Pareto-dominated options to ensure that enough receivers follow the message for senders to have an overriding incentive to choose the message corresponding to their preferred outcome in both the *Lying* and *Hard Truth* conditions^8^.

We use a 2×3 experimental design with two conditions (*Lying* and *Hard Truth*) and three treatments. In the *Baseline* treatment, receivers do not know the payoffs associated with the prosocial and antisocial options, and senders transmit their message anonymously via the computer. This treatment has a similar information structure to the sender-receiver games based on the design of Gneezy (2005). The other treatments are designed to increase the senders' image costs.

In the *Face to Face* treatment, senders deliver the message to the receiver in person. Specifically, senders were asked to write down the message they chose on a blank sheet of paper and wait for an experimenter to come to their desk. The experimenter double-checked that the written message corresponded to the chosen message and then guided the sender to the receiver's desk. The sender handed the sheet over to the receiver and returned to his/her seat. During the delivery process, the experimenter ensured that there was no other communication between senders and receivers.

In the *Face to Face & Information* treatment, in addition to the personal message delivery, the receiver is informed in the instructions of the payoffs available in the 10 options (but stays blind regarding how the computer labels each option)^9^. Note that, since receivers know the payoff structure, we cannot use the same messages as in other treatments because a message stating that an option “will earn you *more money than the other options*, 3 euros” can be immediately identified as a lie during the message delivery. For this reason, we slightly change the wording of the messages of the *Lying* condition. Specifically, Message I reads “Option [letter paying the receiver €10] will earn you 10 euros,” while Message II reads “Option [letter paying the receiver €3] will earn you 10 euros^10^.”

We use the strategy method to measure precisely the senders' willingness to send an antisocial message. Specifically, senders choose between Message I and Message II in each of the 14 rows in Table 2. After that, the computer randomly selects one row to determine which message is sent. When receivers see the message, they are not informed of which row was selected by the computer. While Message I always pays €10, the payoff from Message II equals €17 min the amount *x*, which we systematically vary from €0 to €6.5 in steps of €0.5. Based on the value of *x* at which a sender switches from Message II to Message I, we can calculate the minimum monetary compensation senders must receive to send the antisocial message instead of the prosocial message. In other words, the monetary equivalent of the psychological cost borne by a sender for acting antisocially. Accordingly, we call this minimum compensation the senders' *antisocial cost*. More specifically, senders who choose Message I for all *x* > *c* are classified as having an antisocial cost equal to €6.75−*c* (i.e., the midpoint of the interval [€7−*c*, €6.5−*c*])^11^.

**Table 2:** Senders' choice lists (amounts in euros).

**Row**	**Payoff of**	**x**	**Payoff of**
	**Message I**		**Message II**
1	10.00	0.00	17.00
2	10.00	0.50	16.50
3	10.00	1.00	16.00
4	10.00	1.50	15.50
5	10.00	2.00	15.00
6	10.00	2.50	14.50
7	10.00	3.00	14.00
8	10.00	3.50	13.50
9	10.00	4.00	13.00
10	10.00	4.50	12.50
11	10.00	5.00	12.50
12	10.00	5.50	11.00
13	10.00	6.00	11.00
14	10.00	6.50	10.50

### 2.2. Procedures

We ran the experiment between February and June 2015 at the Laboratory of Experimental Economics (LEE) at University Jaume I in Castellon, Spain, with 240 undergraduate students comprising 121 men and 119 women from different faculties. Participants were recruited using ORSEE (Greiner, 2015). We conducted 12 sessions, each lasting around 1.5 h^12^.

Upon arrival, participants were randomly assigned to computers. After that, the instructions for the experiment were read aloud by the experimenter, and participants were asked to answer a series of control questions (a sample of the instructions is available in the Supplementary Material). Participants could ask questions at any point. The experiment was conducted using z-Tree (Fischbacher, 2007).

Once senders chose a message for each of the 14 values of *x* (see Table 2), the computer randomly selected one of these values and displayed the text of the chosen message on the senders' screen. In the *Face to Face* and *Face to Face & Information* treatments, senders wrote down the message on a sheet of paper and walked over with an experimenter to hand the message over to the receiver. All participants were informed about the delivery process and knew that communication with other participants was forbidden. Once all senders returned to their desks, receivers were asked to type into the computer screen the message they received and choose one of the 10 options.

In addition, we elicited the senders' belief concerning the likelihood that receivers implement the message they receive. Specifically, after the senders delivered their chosen message but before they learned the final outcome, we asked them to indicate “out of 10 Players 2 [the receivers], how many will follow the message they received?” Senders were paid €0.25 for a correct guess^13^.

After the experiment ended, participants were paid in cash. Average earnings were around €15, including belief elicitation and a €5 show-up fee.

### 2.3. Expected Behavior

In line with the literature, we expect to find similar results to Gneezy (2005) in the *Baseline* treatment. Namely, a lower willingness to choose the antisocial message when the message is dishonest than when it is truthful, implying that there are costs to lying. In other words, we expect that the senders' mean antisocial cost is higher in *Lying* than in *Hard Truth*.

The remaining two treatments allow us to test the effects of increasing social image costs on lying and transmitting hard truths. We first introduce social image costs due to the personal delivery of the message in the *Face to Face* treatment, where senders of antisocial messages have to face the receiver in person. In the *Face to Face & Information* treatment, we further increase social image costs because receivers are fully aware of the message's nature and, thus, of the sender's intentions when the message is personally delivered.

The literature shows that social image costs affect behavior in situations with lying (e.g., Gneezy et al., 2018; Bašic and Quercia, 2020) as well as without lying (for social image effects in dictator games see, e.g., Andreoni and Bernheim, 2009; Rigdon et al., 2009; Ockenfels and Werner, 2012)^14^. However, previous work is silent on whether these image costs are greater with or without lying. If the appearance of being dishonest produces larger image costs than that of being willing to transmit a hard truth, then the gap between the *Lying* condition and the *Hard Truth* condition would grow as we move from *Baseline* to *Face to Face*, where the mere physical contact with the receiver might trigger social image concerns, and then to *Face to Face & Information*, where the receiver can also evaluate the actions of the sender. Conversely, if the image costs are stronger in the *Hard Truth* condition than the *Lying* condition, then we would see the treatment differences narrow.

## 3. Results

Our sample consists of 120 receivers and 114 senders: 57 senders in the *Hard Truth* condition (19 senders in each of the three treatments) and 57 senders in the *Lying* condition (19 senders in *Baseline*, 18 in *Face to Face*, and 20 in *Face to Face & Information*)^15^. Descriptive statistics of the main variables per treatment and condition are shown in Supplementary Table 1. We estimate the sample average treatment effects using OLS regressions with robust standard errors. The dependent variable is senders' antisocial cost in section 3.1 and the senders' beliefs about the likelihood that receivers follow the message in section 3.2. The independent variables correspond to treatment and condition dummy variables. The regressions are found in Supplementary Table 2. In addition, we report the results of non-parametric tests. All reported *p*-values are based on two-sided tests.

### 3.1. Senders' Antisocial Cost

**Figure 1:**
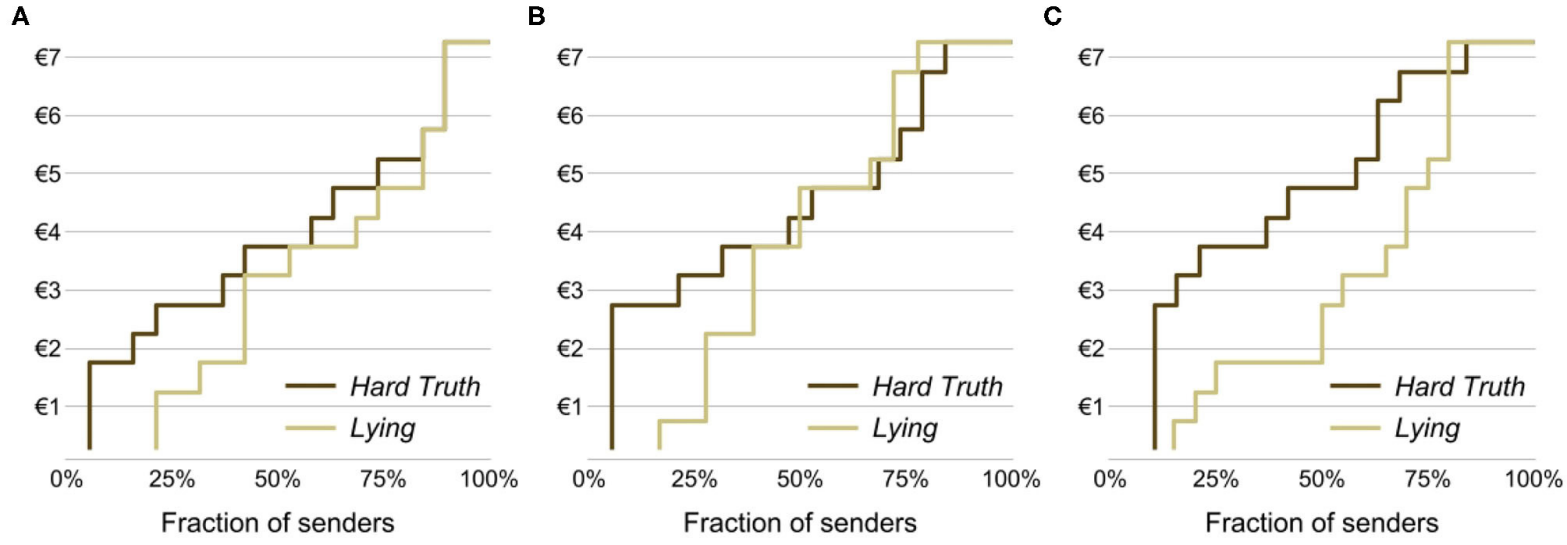
Cumulative distributions of senders' antisocial cost depending on the condition and treatment. **(A)** Baseline. **(B)** Face to Face. **(C)** Face to Face & Information.

**Figure 2:**
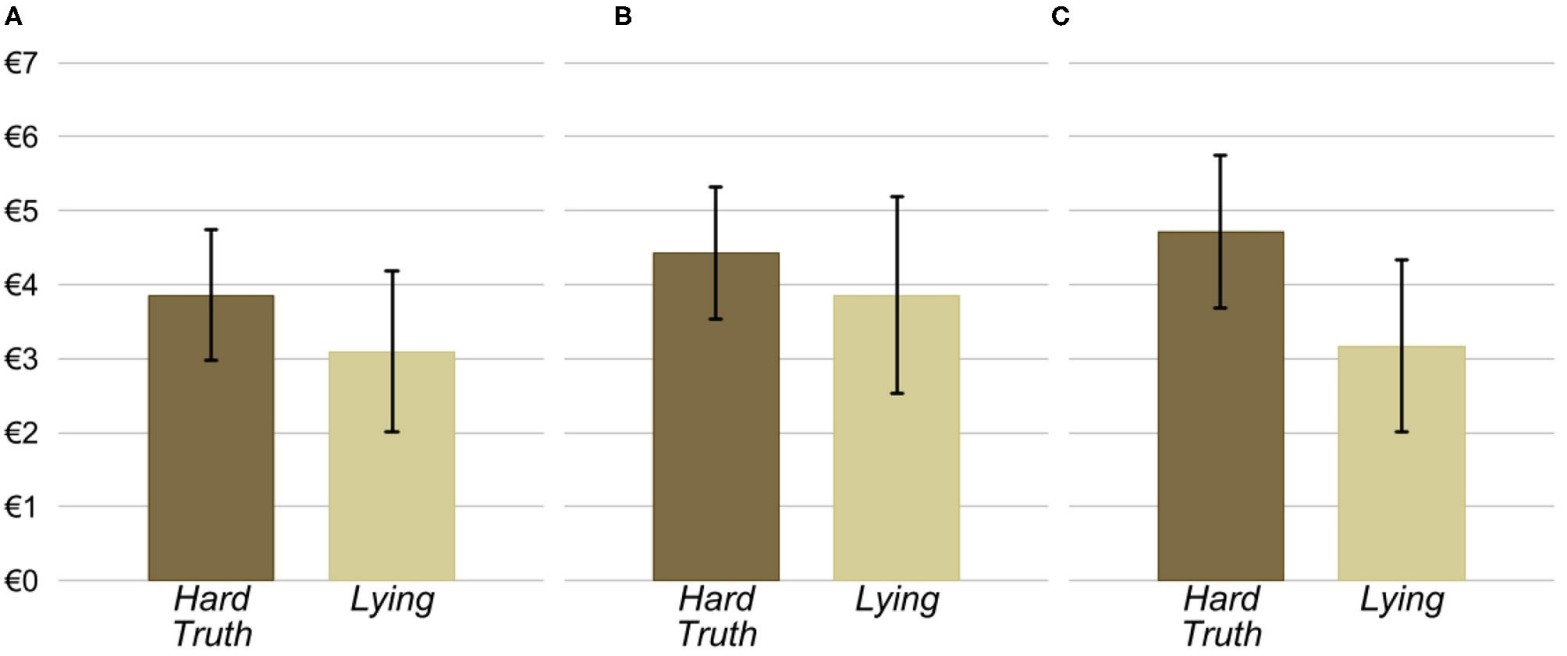
Senders' average antisocial cost and 95% confidence intervals depending on the condition and treatment. **(A)** Baseline. **(B)** Face to Face. **(C)** Face to Face & Information.

Figure 1 depicts the cumulative distributions of the senders' antisocial cost in the *Lying* and *Hard Truth* conditions across the three treatments. Figure 2 shows the senders' average antisocial cost in the two conditions by treatment. These figures suggest that senders are more willing to lie to the receiver than to transmit a hard truth. In fact, pooling observations across the three treatments, we find that the average antisocial cost in the *Lying* condition, €3.36, is significantly lower than the average antisocial cost in the *Hard Truth* condition, €4.34 (*p* = 0.021). The mean difference between conditions is substantial as it corresponds to 0.43 standard deviations^16^.

Next, we look at each treatment separately. In the *Baseline* treatment, we find that, contrary to our expectations, the average antisocial cost is lower in *Lying* than in *Hard Truth* by €0.77 or 0.37 standard deviations. Albeit this difference is not statistically significant (*p* = 0.257). In other words, we do not find evidence that lying induces an additional cost over the cost of acting truthfully but antisocially.

We find a similar result in the *Face to Face* treatment. Namely, a lower average antisocial cost in *Lying* compared to *Hard Truth*. As above, the difference between the two conditions, €0.57 or 0.25 standard deviations, is not statistically significant (*p* = 0.453).

Lastly, we look at the *Face to Face & Information* treatment, where social image costs are presumably highest. As in the other treatments, average antisocial costs are lower in *Lying* than in *Hard Truth*. Unlike the other treatments, at €1.55 or 0.64 standard deviations, this difference is noticeably bigger and statistically significant (*p* = 0.040).

### 3.2. Senders' Beliefs

**Figure 3:**
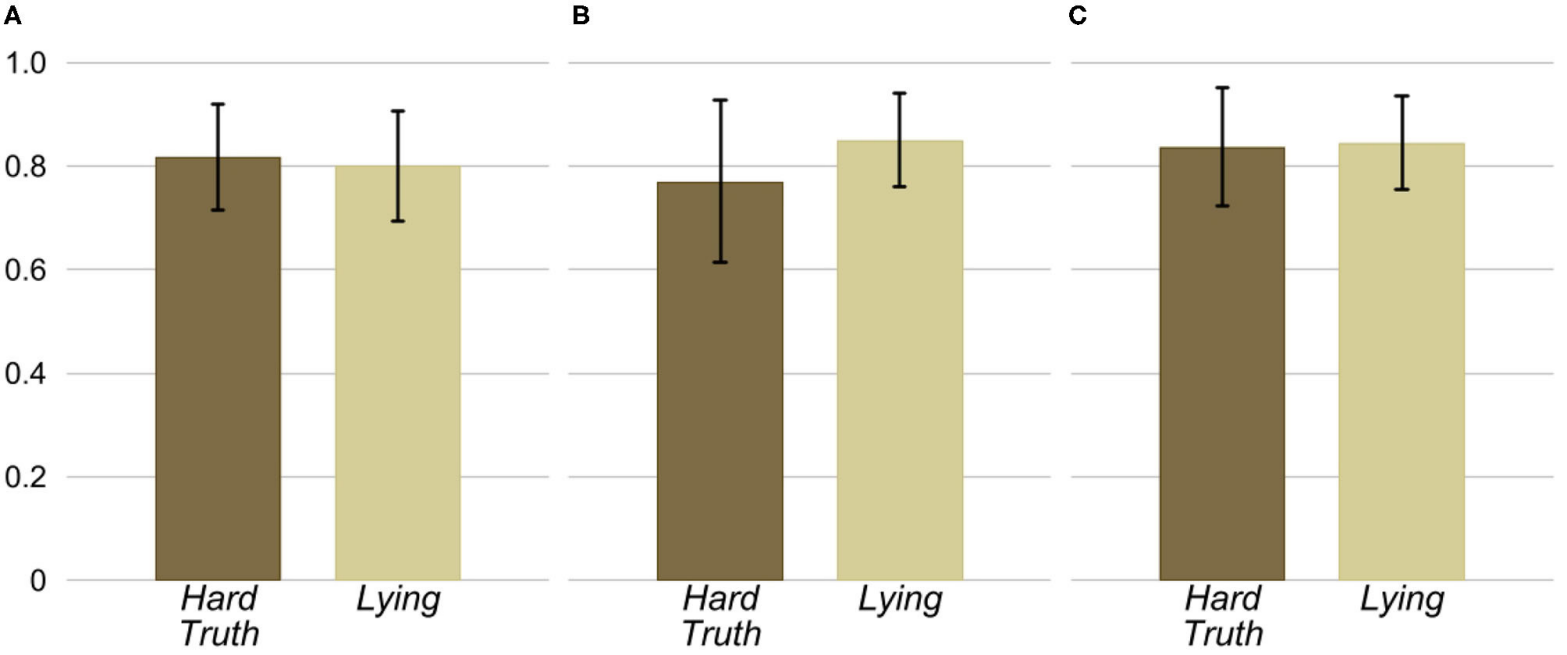
Senders' average belief of the likelihood that a receiver follows the message depending on the condition and treatment. **(A)** Baseline. **(B)** Face to Face. **(C)** Face to Face & Information.

One explanation for the lower willingness to send hard truths than dishonest messages is that senders expect a considerably lower fraction of receivers will follow the message they receive in the *Hard Truth* condition compared to the *Lying* condition. To explore this explanation, we analyze the senders' beliefs about the likelihood that receivers follow the message they receive. The senders' average belief for each condition and treatment is depicted in Figure 3^17^. The figure shows that the average belief is not substantially different across conditions in any of the treatments. Consistent with this observation, we do not find statistically significant differences in the senders' beliefs between the *Hard Truth* and *Lying* conditions in any of the three treatments (*p*>0.353)^18^.

To further check whether the senders' beliefs explain the difference between conditions, we ran additional OLS regressions with the senders' antisocial cost as the dependent variable. As independent variables, we include a dummy variable equal to one if the sender is in the *Lying* condition (and zero otherwise) and the senders' belief (i.e., the fraction of receivers they expect will follow the message). Table 3 contains the regression's estimated coefficients pooling the data from all treatments as well as for each treatment separately. Also, as an additional robustness check, the table includes regressions where we also control for the senders' demographic characteristics (i.e., their gender and age). Overall, the senders' beliefs do not explain the difference between *Lying* and *Hard Truth*^19^.

**Table 3:** Regressions of the senders' antisocial cost on the condition and the senders' belief.

	**All**						**Face to Face**	
	**treatments**		**Baseline**		**Face to Face**		**& Information**	
*Deception* condition	–0.98^*^	–1.02^*^	–0.80	–0.78	–0.74	–0.61	–1.55^*^	–1.76^*^
	(0.42)	(0.42)	(0.67)	(0.68)	(0.75)	(0.74)	(0.76)	(0.78)
Sender's belief	0.35	0.50	–1.90	–1.97	2.03	2.31	-0.01	-0.11
	(0.91)	(0.92)	(1.57)	(1.62)	(1.44)	(1.41)	(1.79)	(1.82)
Demographic controls	No	Yes	No	Yes	No	Yes	No	Yes
Observations	114	114	38	38	37	37	39	39

*OLS estimates and standard errors in parentheses*.

* *Indicates statistical significance at 5%*.

## 4. Conclusions

We investigate under which circumstances an antisocial action that involves a lie could be preferred over an otherwise identical antisocial action that is truthful. We use a series of sender-receiver games in which senders implement a prosocial or an antisocial outcome by sending a prosocial or antisocial message to the receiver. In one condition, the antisocial message involves lying to the receiver, while in the other, the message is truthful. Furthermore, we systematically vary the conditions of the message delivery to vary the social image costs of the sender.

Overall, we do not find evidence in any treatment that lying entails psychological costs above those of acting antisocially. In fact, in the treatment with the highest social image costs, the *Face to Face & Information* treatment, we find the opposite. Senders prefer to implement the antisocial outcome by lying rather than by telling the truth. However, we should note that a potential caveat to this last result is the statistical power of this comparison. An *ex-post* power analysis using the observed means and standard deviations shows that the average treatment effect across the *Lying* and *Hard Truth* conditions in the *Face to Face & Information* treatment has a power of 0.52 for a significance level of 5%. Therefore, it would be premature to conclude that the psychological costs of lying are *lower* than those of telling a hard truth. Future work ought to gather more evidence to substantiate this effect. Having said that, the fact that in all three treatments, the senders' antisocial costs of implementing the antisocial outcome by lying are never higher than those of implementing the same outcome with a truthful message shows more convincingly that the willingness to lie is sensitive to the image costs of the no-lying alternative.

We think that our experiment highlights the need to understand the impact of social image costs on different decisions. In settings where actions have consequences for others, social image costs are present irrespective of whether the antisocial action involves lying or not. Hence, the social image cost of being perceived as dishonest needs to be compared to the social image cost of being perceived as someone willing to deliver hard or uncomfortable truths. Our results suggest that the discomfort experienced when delivering an antisocial message in person when the recipient can immediately interpret the message's content is higher than that of eventually being perceived as dishonest.

Our setup suggests that it is important to consider the timing of social contact and the moment when others learn the nature of one's actions, which is when they can judge them as good or bad. The personal delivery of the message when receivers are fully informed implies that an antisocial truthful message can be judged as bad at the moment of social contact. This simultaneity could make social image costs more salient. By contrast, a dishonest antisocial message will not be judged immediately but later on when the receiver learns the implemented message's outcome. This separation in time allows the sender to “hide behind the lie” at the moment of social contact. Therefore, even if the sender knows that the message will eventually be revealed as a lie, the social image cost of appearing dishonest occurs at a point where social image costs are likely to be less salient. We think this last result merits further study. We find that the antisocial costs of lying are substantially lower than those of telling a hard truth in the *Face to Face & Information* treatment, which supports this interpretation. However, we also find a smaller difference in the same direction in the *Face to Face* treatment^20^. Given that in the *Face to Face* the hard truth message does not reveal one's intentions, there can be reasons other than “hiding” to prefer a lie over a hard truth.

## Data Availability Statement

The data for this study is available at https://doi.org/10.3886/E143161V1. Replication materials are included in the Supplementary Material, further inquiries can be directed to the corresponding author/s.

## Ethics Statement

The studies involving human participants were reviewed and approved by IRB Columbia University (Protocol IRB-AAAO9551). The participants provided their written informed consent to participate in this study.

## Author Contributions

All authors listed have made a substantial, direct and intellectual contribution to the work, and approved it for publication.

## Conflict of Interest

The authors declare that the research was conducted in the absence of any commercial or financial relationships that could be construed as a potential conflict of interest.

## Footnotes

^1^See, for example, Lundquist et al. (2009), Kartik (2009), Erat and Gneezy (2012), Cappelen et al. (2013), Gibson et al. (2013), Gneezy et al. (2013), López-Pérez and Spiegelman (2013), Abeler et al. (2014), Kajackaite and Gneezy (2017), Gneezy et al. (2018), and Alempaki et al. (2019). Moreover, see Tang et al. (2018) and Abeler et al. (2019) for meta-analyses of the literature and Sobel (2020) for a discussion on the distinction between lying and deception in games.

^2^This finding was later replicated by Hurkens and Kartik (2009) using the same design. Gneezy (2005) also shows that senders react to different monetary consequences of lying for the sender and the receiver. Hurkens and Kartik (2009) identify two types of individuals in this setting, those who never lie and those who lie whenever the monetary benefit from lying is preferred over being truthful.

^3^See Bartling and Fischbacher (2012) for evidence that delegated decisions reduce the responsibility of a decision-maker for an antisocial outcome, even when the player to which the decision is delegated has strong incentives to act as the original decision-maker intended.

^4^Conrads and Lotz (2015) find that individuals lie less in face-to-face settings than in more anonymous settings.

^5^We designed the sender-receiver games so that almost all receivers implement the option mentioned in the sender's message irrespective of the message's content and whether they are informed of the payoff structure or not. In other words, we ensure that there are no strategic reasons for senders to send a message that does not correspond to the outcome they would like to see implemented. See section 3 for details.

^6^According to Sobel (2020), while *Hard Truth* messages do not involve lying, they might be deceptive depending on the receivers' beliefs and available information. One could argue that this is the case in the *Baseline* and *Face to Face* treatments, where receivers might be lead to think that the sender is acting in their best interest, but not in the *Face to Face & Information* treatment.

^7^These messages are based on those used by Gneezy (2005). In that paper, the lying message was “Option B will earn you more money than option A,” when in fact, A paid the receiver more than B. Since we had more than two options, we used “other options” instead of “option A.” Moreover, we added the amount in euros to make the message in the *Lying* condition comparable to that in the *Hard Truth* condition.

^8^Specifically, we chose monetary payoffs so that senders have a strict incentive to send the message corresponding to their preferred outcome as long as they expect more than 10% of the receivers to follow their message. To see this, denote the sender's utility if the antisocial option is implemented as *U*(*A*), her utility if the prosocial option is implemented as *U*(*P*), and her utility if a dominated option is implemented as *U*(*D*). Furthermore, let *b*∈[0, 1] be the sender's belief that the receiver follows the message. In this case, the sender's expected utility of sending Message I is *bU*(*P*) + (1−*b*)(1/9)*U*(*A*) + (1−*b*)(8/9)*U*(*D*), and that of sending Message II is *bU*(*A*) + (1−*b*)(1/9)*U*(*P*) + (1−*b*)(8/9)*U*(*D*). It follows that as long as *b*>1/10, senders will choose Message I if *U*(*P*)>*U*(*A*) and Message II if *U*(*P*) < *U*(*A*). Note that this condition holds for 97.4% of the senders in our dataset.

^9^In all treatments, it is common knowledge that a message always reveals the label of either the prosocial or the antisocial option and never the label of one of the Pareto-dominated options.

^10^This difference implies a slight change in the nature of the lie between treatments. While in *Baseline* and *Face to Face* the sender lies about an option paying the receiver “more money than the other options,” in *Face to Face & Information*, the sender lies about the stated amount “10 euros,” which the receiver knows would pay more than other options. An alternative experimental design would be to use the messages from *Face to Face & Information* in all treatments. However, that would make those treatments less comparable to Gneezy (2005), which is why we opted for our current design.

^11^At the extremes, senders who always choose Message I are classified as having an antisocial cost equal to €7.25 and senders who always choose Message II as having equal to €0.25. Senders who switched more than once or switched from Message I to Message II.

^12^Data from the *Face to Face* treatments are also used in Behnk et al. (2019). Data from the *Baseline* and *Face to Face & Information* treatments are exclusively used in this study.

^13^We also elicited participants' normative views and their beliefs about normative views of others. Furthermore, we elicited the receivers' expected fraction of antisocial messages. A rigorous analysis of these variables in the *Face to Face* treatment is reported in Behnk et al. (2019).

^14^Importantly, this research has demonstrated that social image costs can be triggered even in anonymous settings where, logically, their social image should not be a concern (Gneezy et al., 2018). Another interpretation of these results is that people also care about self-image. In other words, they want to signal to themselves through their actions that they are a prosocial individual (Bénabou and Tirole, 2006).

^15^Of the 120 senders, six senders switched more than once between Message I and Message II in the choice list. Since it is not clear what these participants' antisocial cost is, we dropped them from the statistical analysis.

^16^The *p*-values of comparing the distribution of antisocial costs across the two conditions using Wilcoxon-Mann-Whitney *U*-tests are as follows: *p* = 0.026 pooling across treatments; *p* = 0.259 in *Baseline*; *p* = 0.612 in *Face to Face*; *p* = 0.058 in *Face to Face & Information*.

^17^The actual fraction of receivers who follow the message they receive equals 0.98 in *Lying* (0.95 in *Baseline*, 1.00 in *Face to Face*, and 1.00 in *Face to Face & Information*) and 0.84 in *Hard Truth* (0.90 in *Baseline*, 0.84 in *Face to Face*, and 0.77 in *Face to Face & Information*). Hence, senders' are somewhat pessimistic about the receivers following rate.

^18^The *p*-values of comparing the distribution of the senders' beliefs across the two conditions using Wilcoxon-Mann-Whitney *U*-tests are as follows: *p* = 0.919 pooling across treatments; *p* = 0.714 in *Baseline*; *p* = 0.895 in *Face to Face*; *p* = 0.999 in *Face to Face & Information*.

^19^The senders' beliefs are not statistically significant in any of the regressions. This result is to be expected given that most beliefs are relatively high, and a very low belief is required for it to be relevant to the sender's choice (see Footnote 8).

^20^A difference-in-difference test of the average effect of the condition results in an insignificant result (*p* = 0.362).
