# Supplementary material for: On Lies and Hard Truths
Source: Front Psychol. 2021 Jul 7;12:687913. doi: 10.3389/fpsyg.2021.687913 (PMC8292950; doi:10.3389/fpsyg.2021.687913)
Supplement: Supplementary file 1 [file Data_Sheet_1.PDF]

On lies and hard truths

Supplementary Material

1 Additional statistical analyses

Table A1 shows the mean and standard deviations of the senders' antisocial cost and their belief of the likelihood that the receiver follows the message. The statistics are shown first by condition and then by treatment and condition.

Table A1. Means and standard deviations (in parenthesis) of the senders' antisocial cost and their belief of the likelihood that the receiver follows the message

	Antisocial cost (in euros)	Expected likelihood that the receiver follows the message	Number of observations
Lying (all)	3.36	0.83	57
	(2.47)	(0.20)	
Hard Truth (all)	4.34	0.81	57
	(1.95)	(0.26)	
Lying + Baseline	3.09	0.80	19
	(2.27)	(0.22)	
Hard Truth + Baseline	3.86	0.82	19
	(1.84)	(0.21)	
Lying + Face to Face	3.86	0.85	18
	(2.68)	(0.18)	
Hard Truth + Face to Face	4.43	0.77	19
	(1.86)	(0.33)	
Lying + Face to Face & Information	3.17	0.85	20
	(2.50)	(0.20)	
Hard Truth + Face to Face & Information	4.72	0.84	19
	(2.15)	(0.24)	

Table A2, contains the regressions used to estimate the sample average treatment effects and evaluate their statistical significance. The coefficients are estimated using linear regressions with robust standard errors. Quantitively similar results are obtained with bootstrapped standard errors.

Table A2. Average treatment differences in senders' antisocial cost and their belief of the likelihood that the receiver follows the message

Note: Linear regressions of the senders' antisocial cost (models I and II) and their belief of the likelihood of the receiver following the message (models III and IV). Robust standard errors in parentheses. ***, **, and * indicate statistical significance at 0.01, 0.05, and 0.10.

	Senders' Antisocial cost		Expected likelihood of the receiver following the message	
	I	II	III	IV
Lying	–0.97**		0.02	
	(0.42)		(0.04)	
Face to Face		0.58		–0.05
		(0.60)		(0.09)
Face to Face & Information		0.87		0.02
		(0.65)		(0.07)
Baseline × Lying		–0.76		–0.02
		(0.67)		(0.07)
Face to Face × Lying		–0.57		0.08
		(0.76)		(0.09)
Face to Face & Information × Lying		–1.55**		0.01
		(0.75)		(0.07)
Constant	4.33***	3.86***	0.81***	0.82***
	(0.26)	(0.42)	(0.03)	(0.05)
Observations	114	114	114	114
F-statistic	5.46	1.69	0.29	0.28
R^2	0.05	0.07	0.00	0.01

2 Experimental instructions

This section contains the experimental instructions used for the senders in the *Face to Face* treatment in the *Lying* condition. Differences with the *Hard Truth* condition are highlighted in red (screenshots contain the text from the *Lying* condition). All instructions are translated from Spanish. Instructions for the *Baseline* and *Face to Face & Information* treatments are very similar and available upon request.

General instructions

You are participating in a study on economic decision-making. You have already earned **€5** for showing up on time. Please read these instructions carefully as they describe how you can earn **additional** money. You will be paid all your earnings in cash.

Please do not talk or communicate with other participants in any way. If you have questions, raise your hand and one of us will help you.

In the study, all participants are randomly assigned to groups of two. None of you will know the identity of the other player at any time. Within each group, the computer randomly assigns participants to the roles of **Player 1** and **Player 2**. You will be informed of your role on the computer screen.

Summary of the study

- There are ten options with payments for each player. Player 1 is informed of the payment each player receives in each option. On the other hand, Player 2 does not receive this information.
- **Player 1 chooses one message out of the two available messages** to be sent to Player 2. [Each message states that a specific option is the option that gives the highest payment to Player 2.] [Each message states the payment that a specific option gives to Player 2.]
- Player 1 delivers the message to Player 2 in person.
- **Player 2 chooses an option** that determines the earnings of both players.

Specific instructions for Player 1

There are ten options, each labeled with a unique letter: A, B, C, D, E, F, G, H, I, or J. The computer will randomly assign one option to pay **€10 to Player 1 and €10 to Player 2** and another option to pay **€17 to Player 1 and €3 to Player 2**. The remaining eight options pay **€4 to Player 1 and €0 to Player 2**.

How much each player earns in each option will be shown only to Player 1. The following table is an example of how payments could be assigned to the various options and how this information would be presented to Player 1.

**Option**	A	B	**C**	D	**E**	F	G	H	I	J
Player 1's payment	4	4	10	4	17	4	4	4	4	4
Player 2's payment	0	0	10	0	3	0	0	0	0	0

Player 2 knows that there are ten options and that among them there are 8 options that pay €4 to Player 1 and €0 to Player 2. However, Player 2 neither knows that there is an option that pays €10 to Player 1 and €10 to Player 2, nor that there is another option that pays €17 to Player 1 and €3 to Player 2. **Player 2 only knows that there are two options with positive payoffs for both players. Finally, Player 2 does not know how the computer assigned the payments to the options.**

The table below shows what Player 2 will see.

Option	A	B	C	D	E	F	G	H	I	J
Player 1's payment	?	?	?	?	?	?	?	?	?	?
Player 2's payment	?	?	?	?	?	?	?	?	?	?

The only information that Player 2 receives regarding the payments of the various options is the message chosen and sent by Player 1. After receiving the message, Player 2 chooses one of the ten options. The option chosen by Player 2 determines the earnings of both players in the group.

Player 1 chooses a message

Player 1 chooses **one** message for Player 2. There are **two available messages**. Each message corresponds to one of the two options with positive earnings for both players.

- [**Message I** corresponds to the option that pays €10 to Player 2. The message reads "Option <*letter of option that pays €10 to Player 2*> will earn you more money than the other options, 10 euros".]
- [**Message I** corresponds to the option that pays €10 to Player 2. The message reads "Option <*letter of option that pays €10 to Player 2*> will earn you 10 euros".]
- [**Message II** corresponds to the option that pays €3 to Player 2. The message reads "Option <*letter of option that pays €3 to Player 2*> will earn you more money than the other options, 3 euros".]
- [**Message II** corresponds to the option that pays €3 to Player 2. The message reads "Option <*letter of option that pays €3 to Player 2*> will earn you 3 euros".]

Note that Player 1 cannot choose a message that corresponds to an option that pays €0 to Player 2.

[Therefore, when Player 2 receives a message, he/she will not know whether the option mentioned in the message is the one that will pay him/her more money, but he/she can be certain that the option does not pay him/her €0.]

[Therefore, when Player 2 receives Message I, he/she will know the letter of the option that pays him/her €10, but he/she will not know which is the other option with a positive payment for him/her, nor will he/she know that this option pays him/her €3.

In the same way, if Player 2 receives Message II, he/she will know the letter of the option that pays him/her €3, but he/she will not know which is the other option with a positive payment for him/her, nor will he/she know that this other option pays him/her €10.]

Example

Suppose that the computer randomly assigns payments to options as shown in the table below.

**Option**	A	B	C	**D**	E	**F**	G	H	I	J
Player 1's payment	4	4	4	17	4	10	4	4	4	4
Player 2's payment	0	0	0	3	0	10	0	0	0	0

In this case, Player 2 can receive one of the following two messages:

["Option F will earn you more money than the other options, 10 euros"]

["Option F will earn you 10 euros"]

["Option D will earn you more money than the other options, 3 euros"]

["Option D will earn you 3 euros"]

Player 1 sends the message to Player 2. If the option mentioned in the message coincides with the option subsequently chosen by Player 2, then **Player 1 pays a cost between €0 and €6.50 for sending the message**. The screens below will be used to determine which message Player 1 sends and how much Player 1 pays. Each screen displays a list containing 14 rows, each row representing a possible cost. Player 1 must decide between Message I and Message II in each of the 14 rows.

Decisions of Player 1

You are Player 1

**Option**	A	B	C	D	E	F	G	H	I	J
Player 1's payment	10	4	4	4	4	4	4	17	4	4
Player 2's payment	10	0	0	0	0	0	0	3	0	0

Please decide between Message I and Message II in **each row.**

Message I:
Option A will earn you more money than the other options, 10 euros

Player 2 earns **€ 10** if he/she chooses Option A.

Message II:
Option H will earn you more money than the other options, 3 euros

Player 2 earns **€ 3** if he/she chooses Option H.

**Row**	**Cost**	**Your payment**		**Cost**	**Your payment**
1	0	10	○ ○	0.0	17.0
2	0	10	○ ○	0.5	16.5
3	0	10	○ ○	1.0	16.0
4	0	10	○ ○	1.5	15.5
5	0	10	○ ○	2.0	15.0
6	0	10	○ ○	2.5	14.5
7	0	10	○ ○	3.0	14.0
8	0	10	○ ○	3.5	13.5
9	0	10	○ ○	4.0	13.0
10	0	10	○ ○	4.5	12.5
11	0	10	○ ○	5.0	12.0
12	0	10	○ ○	5.5	11.5
13	0	10	○ ○	6.0	11.0
14	0	10	○ ○	6.5	10.5

Specifically, in each row, Player 1 decides between:

- Choosing **Message I** and paying a cost of **€0** for sending this message.
- Choosing **Message II** and paying a cost **specified in that row** for sending this message.

After Player 1 has made his/her decisions, **one of the 14 rows will be randomly selected** by the computer to determine which message will be sent to Player 2. All rows have the same chance of being

selected; therefore, you should make your decision in each row seriously. Player 1 will send the message determined by his/her choices in the selected row in the following way:

- In the selected row, if **Player 1 chose Message I**, then **Player 1 sends Message I**. In this case, if Player 2 chooses the option corresponding to Message I, then both Player 1 and Player 2 earn €10.
- In the selected row, if **Player 1 chose Message II**, then **Player 1 sends Message II**. In this case, if Player 2 chooses the option corresponding to Message II, then Player 1 earns €17 minus the cost specified in that row and Player 2 earns €3.

Player 2 will **not** be informed which row was selected by the computer.

Examples

Suppose that Player 1 makes the choices shown below.

Decisions of Player 1

You are Player 1

Option	A	B	C	D	E	F	G	H	I	J
Player 1's payment	10	4	4	4	4	4	4	17	4	4
Player 2's payment	10	0	0	0	0	0	0	3	0	0

Please decide between Message I and Message II in **each row.**

Message I:
Option A will earn you <u>more money than the other options</u>, 10 euros
Player 2 earns **€ 10** if he/she chooses Option A.

Message II:
Option H will earn you <u>more money than the other options</u>, 3 euros
Player 2 earns **€ 3** if he/she chooses Option H.

Row	Cost	Your payment		Cost	Your payment
1	0	10	○ ●	0.0	17.0
2	0	10	○ ●	0.5	16.5
3	0	10	○ ●	1.0	16.0
4	0	10	○ ●	1.5	15.5
5	0	10	○ ●	2.0	15.0
6	0	10	○ ●	2.5	14.5
7	0	10	○ ●	3.0	14.0
8	0	10	○ ●	3.5	13.5
9	0	10	○ ●	4.0	13.0
10	0	10	○ ●	4.5	12.5
11	0	10	● ○	5.0	12.0
12	0	10	● ○	5.5	11.5
13	0	10	● ○	6.0	11.0
14	0	10	● ○	6.5	10.5

In this example, Player 1 is willing to pay at maximum €4.5 for sending Message II. Given these choices, the following occurs if the computer randomly selects one of the rows below:

- **Row 9:** Since Player 1 chose Message II then he/she sends Message II. Thereafter, if Player 2 chooses the option corresponding to Message II, then Player 1 earns €17 – €4 = €13 and Player 2 earns €3.
- **Row 12:** Since Player 1 chose Message I then he/she sends Message I. Thereafter, if Player 2 chooses the option corresponding to Message I, then both Player 1 and Player 2 earn €10.

Suppose that Player 1 makes the choices shown below.

Decisions of Player 1

You are Player 1

Option	A	B	C	D	E	F	G	H	I	J
Player 1's payment	10	4	4	4	4	4	4	17	4	4
Player 2's payment	10	0	0	0	0	0	0	3	0	0

Please decide between Message I and Message II in **each row.**

Message I:
Option A will earn you more money than the other options, 10 euros
Player 2 earns **€ 10** if he/she chooses Option A.

Message II:
Option H will earn you more money than the other options, 3 euros
Player 2 earns **€ 3** if he/she chooses Option H.

Row	Cost	Your payment		Cost	Your payment
1	0	10	○ ●	0.0	17.0
2	0	10	○ ●	0.5	16.5
3	0	10	○ ●	1.0	16.0
4	0	10	○ ●	1.5	15.5
5	0	10	○ ●	2.0	15.0
6	0	10	● ○	2.5	14.5
7	0	10	● ○	3.0	14.0
8	0	10	● ○	3.5	13.5
9	0	10	● ○	4.0	13.0
10	0	10	● ○	4.5	12.5
11	0	10	● ○	5.0	12.0
12	0	10	● ○	5.5	11.5
13	0	10	● ○	6.0	11.0
14	0	10	● ○	6.5	10.5

In this example, Player 1 is willing to pay at maximum €2 for sending Message II. Given these choices, the following occurs if the computer randomly selects one of the rows below:

- **Row 4:** Since Player 1 chose Message II then he/she sends Message II. Thereafter, if Player 2 chooses the option corresponding to Message II, then Player 1 earns €17 – €1.5 = €15.5 and Player 2 earns €3.
- **Row 6:** Since Player 1 chose Message I then he/she sends Message I. Thereafter, if Player 2 chooses the option corresponding to Message I, then both Player 1 and Player 2 earn €10.

Player 1 delivers the message to Player 2 in person

Once the message is determined, Player 1 will see a screen like the one below.

Please **write down the following message** on the sheet of paper located on your desk and **wait until an experimenter arrives.**

The experimenter will check that the message you wrote down coincides with the message below.

Option J will earn you more money than the other options, 10 euros

To deliver the message, Player 1 will first **write down the message on the sheet of paper** located on his/her desk. Then, Player 1 will wait until an experimenter arrives. The experimenter will check whether the message written on the sheet of paper is identical to the message shown on the screen. Note that, like Player 2, the experimenter will not know to which payment the option in the message corresponds.

The experimenter will then walk with Player 1 to the desk of the Player 2 of his/her group. At this point, Player 1 will hand the paper with the message to Player 2 and then walk back to his/her desk.

Remember that **any kind of communication between the players is prohibited**, including gestures and facial expressions. In addition, Player 1 is not allowed to write down anything else other than the message on the sheet of paper. Any participant who does not comply with these rules will not be paid at the end of the study.

Player 2 chooses an option

Player 2 knows that there are two options with positive payments for him/her, but he/she neither knows to which amounts of money these payments are associated, nor which of the ten options contain these positive payments.

[The only information that Player 2 receives is the message sent to him/her by Player 1.]

[The only information that Player 2 receives is the message, stating the payment that a specific option pays her, sent to him/her by Player 1.]

After receiving the message, Player 2 sees a screen like this:

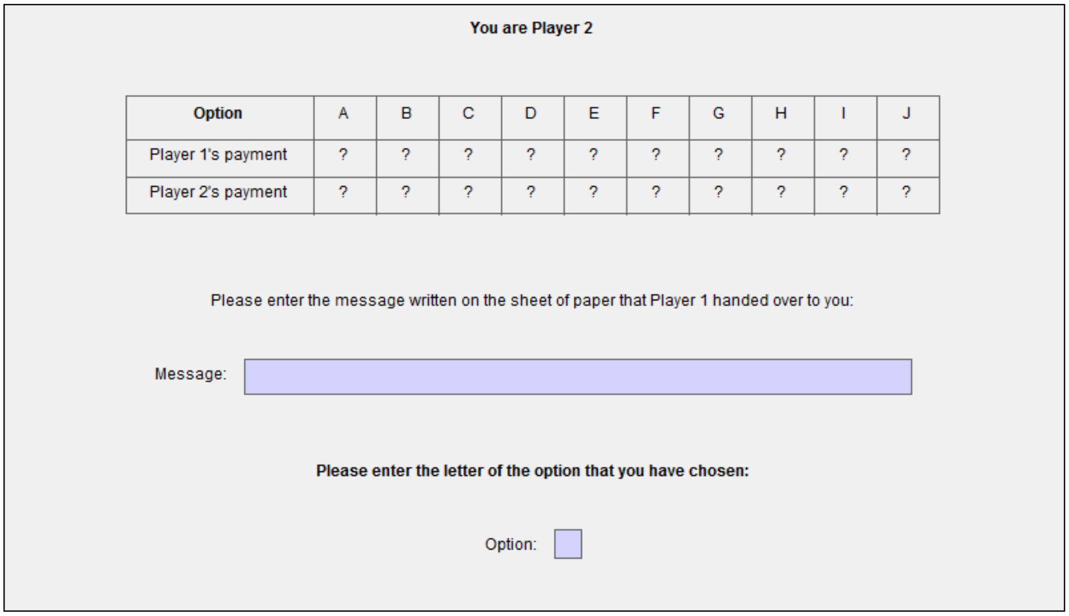

On this screen, Player 2 first confirms the message he/she received by typing it into the text box. Then, he/she chooses one of the ten options. **The option chosen by Player 2 determines the earnings of all players**. Remember that if Player 2 chooses a zero-payment option, the final earnings will be €0 for him/her and 4€ for Player 1.

Specific instructions for Player 2

There are ten options, each labelled with a unique letter: A, B, C, D, E, F, G, H, I, or J. Eight out of the ten options randomly assigned by the computer pay **€4 to Player 1 and €0 to Player 2**. The other two options contain **positive payoffs for each player** (in other words, they pay more than €0). Players 1 knows all payments and also knows **how the computer assigned the payments to the ten options**.

Player 2 neither knows the payments of the two options with positive payments nor how the computer assigned the payments to the options. The table below shows what Player 2 will see.

Option	A	B	C	D	E	F	G	H	I	J
Player 1's payment	?	?	?	?	?	?	?	?	?	?
Player 2's payment	?	?	?	?	?	?	?	?	?	?

The only information that Player 2 receives regarding the payments of the various options is the message chosen and sent by Player 1. After receiving the message, Player 2 chooses one of the ten options. The option chosen by Player 2 determines the earnings of both players in the group.

Player 1 chooses a message and sends it to Player 2

Player 1 chooses **one message** for Player 2. There are **two available messages**. Each message corresponds to one of the two options with positive earnings for both players.

- [Message I reads "Option <letter of option with highest positive payment for Player 2> will earn you more money than the other options, <highest positive payment> euros".]
- [Message I reads "Option <letter of option with highest positive payment for Player 2> will earn you <highest positive payment> euros".]
- [Message II reads "Option [letter of option with lowest positive payment for Player 2] will earn you more money than the other options, <lowest positive payment> euros".]
- [Message II reads "Option [letter of option with lowest positive payment for Player 2] will earn you <lowest positive payment> euros".]

Note that Player 1 cannot choose a message that corresponds to an option that pays €0 to Player 2. Therefore, when Player 2 receives a message, he/she will not know whether the option mentioned in the message pays him/her the highest positive payment, but he/she can be certain that the option does not pay him/her €0.

Player 1 must decide between Message I and Message II. Specifically, Player 1 decides between:

- Choosing **Message I** and paying a cost of **€0** for sending this message.
- Choosing **Message II** and paying a **positive amount of money** for sending this message.

Player 2 will **not** be informed which cost Player 1 paid.

Player 1 delivers the message to Player 2 in person

Once the message is determined, Player 1 will see a screen like the one below.

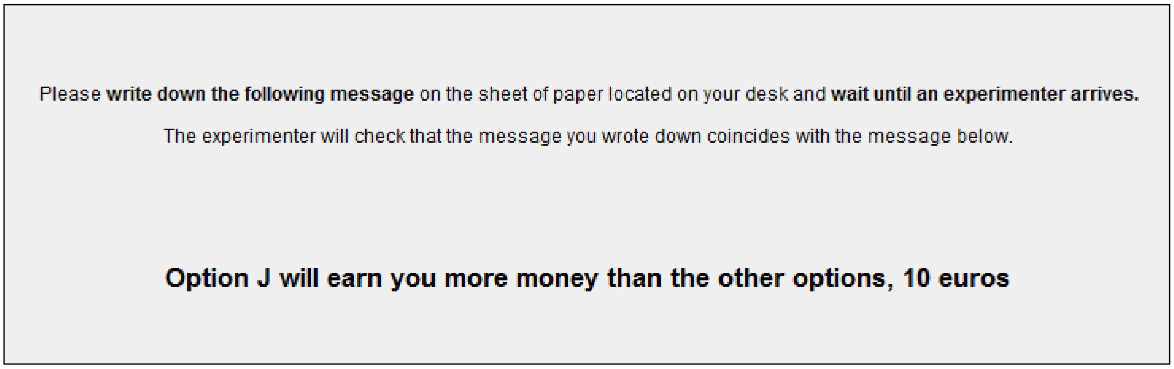

To deliver the message, Player 1 will first **write down the message on the sheet of paper** located on his/her desk. Then, Player 1 will wait until an experimenter arrives. The experimenter will check whether the message written on the sheet of paper is identical to the message shown on the screen. Note that, like Player 2, the experimenter will not know to which payment the option in the message corresponds.

The experimenter will then walk with Player 1 to the desk of the Player 2 of his/her group. At this point, Player 1 will hand the paper with the message to Player 2 and then walk back to his/her desk.

Remember that **any kind of communication between the players is prohibited**, including gestures and facial expressions. In addition, Player 1 is not allowed to write down anything else other than the message on the sheet of paper. Any participant who does not comply with these rules will **not be paid** at the end of the study.

Player 2 chooses an option

Player 2 knows that there are two options with positive payments for him/her, but he/she neither knows to which amounts of money these payments are associated, nor which of the ten options contain the positive payments.

[The only information that Player 2 receives is the message sent to him/her by Player 1.]

[The only information that Player 2 receives is the message, stating the payment that a specific option pays her, sent to him/her by Player 1.]

After receiving the message, Player 2 sees a screen like the one below.

On this screen, Player 2 first confirms the message he/she received by typing it into the text box. Then, he/she chooses one of the ten options. **The option chosen by Player 2 determines the earnings of both players**. Remember that if Player 2 chooses a zero-payment option, the final earnings will be €0 for him/her and 4€ for Player 1.

You are Player 2

Option	A	B	C	D	E	F	G	H	I	J
Player 1's payment	?	?	?	?	?	?	?	?	?	?
Player 2's payment	?	?	?	?	?	?	?	?	?	?

Please enter the message written on the sheet of paper that Player 1 handed over to you:

Message:

Please enter the letter of the option that you have chosen:

Option:
